# Subsequent and simultaneous electrophysiological investigation of the retina and the visual cortex in neurodegenerative and psychiatric diseases: what are the forecasts for the medicine of tomorrow?

**DOI:** 10.3389/fpsyt.2023.1167654

**Published:** 2023-06-02

**Authors:** Katelyne Tursini, Irving Remy, Steven Le Cam, Valérie Louis-Dorr, Hélène Malka-Mahieu, Raymund Schwan, Grégory Gross, Vincent Laprévote, Thomas Schwitzer

**Affiliations:** ^1^Pôle Hospitalo-Universitaire de Psychiatrie d’Adultes et d’Addictologie du Grand Nancy, Centre Psychothérapique de Nancy, Laxou, France; ^2^BioSerenity, Paris, France; ^3^INSERM U1254, Université de Lorraine, IADI, Nancy, France; ^4^INSERM U1114, Université de Strasbourg, Strasbourg, France; ^5^CRAN, CNRS UMR 7039, Université de Lorraine, Nancy, France; ^6^Faculté de Médecine, Université de Lorraine, Vandœuvre-lès-Nancy, France

**Keywords:** ERG = electroretinogram, EEG, VEP = visual evoked potential, psychiatry, neurodegenerative disease, retina, visual cortex (V1)

## Abstract

Visual electrophysiological deficits have been reported in neurodegenerative disorders as well as in mental disorders. Such alterations have been mentioned in both the retina and the cortex, notably affecting the photoreceptors, retinal ganglion cells (RGCs) and the primary visual cortex. Interestingly, such impairments emphasize the functional role of the visual system. For this purpose, the present study reviews the existing literature with the aim of identifying key alterations in electroretinograms (ERGs) and visual evoked potentials electroencephalograms (VEP-EEGs) of subjects with neurodegenerative and psychiatric disorders. We focused on psychiatric and neurodegenerative diseases due to similarities in their neuropathophysiological mechanisms. Our research focuses on decoupled and coupled ERG/VEP-EEG results obtained with black-and-white checkerboards or low-level visual stimuli. A decoupled approach means recording first the ERG, then the VEP-EEG in the same subject with the same visual stimuli. The second method means recording both ERG and VEP-EEG simultaneously in the same participant with the same visual stimuli. Both coupled and decoupled results were found, indicating deficits mainly in the N95 ERG wave and the P100 VEP-EEG wave in Parkinson’s, Alzheimer’s, and major depressive disorder. Such results reinforce the link between the retina and the visual cortex for the diagnosis of psychiatric and neurodegenerative diseases. With that in mind, medical devices using coupled ERG/VEP-EEG measurements are being developed in order to further investigate the relationship between the retina and the visual cortex. These new techniques outline future challenges in mental health and the use of machine learning for the diagnosis of mental disorders, which would be a crucial step toward precision psychiatry.

## 1. Introduction

Recent advances in neuroscience and psychiatry focus on the search and the development of relevant indicators that can accurately reflect brain condition in pathological states such as in neurodegenerative and psychiatric disorders. These indicators need to be sensitive to the pathophysiological processes and damage underlying neurodegenerative such as Parkinson and Alzheimer diseases as well as psychiatric disorders such as schizophrenia and bipolar disorders. Furthermore, both types of illnesses displayed dysfunctions in neurotransmission, inflammation, autoimmunity, and neurodegeneration (1). Of all the areas of interest, the use of visual electrophysiological methods to evaluate visual function is of particular relevance. Visual electrophysiology, including retinal and cortical measurements, provides information about the functional properties of neural networks in the visual pathway from the retina to the brain. Visual pathway neurons display similarities to brain neurons in terms of function, damage, and response. Thus, examining the neural functioning of the visual pathway can help in the understanding of pathophysiological brain conditions including psychiatric disorders. Accordingly, the next part of this review will describe the potential value and the evidence for combining several electrophysiological indicators.

## 2. Potential value and evidence for combining several electrophysio- logical indicators

### 2.1. Anatomy of the visual pathway and relevant indicators for investigating neuropsychiatric disorders

The study of visual function is of particular interest for several reasons. Interestingly, both retina and brain share the same neurodevelopmental origin at embryological stage. Indeed, both structures have the same embryonic origin and derive from the diencephalon (2). Moreover, the retina and the visual cortex share an anatomical link with the optic nerve (3). The retina and the brain also share the same main neurotransmitters, such as glutamate, dopamine, serotonin, and *γ*-aminobutyric acid (GABA) (4). Interestingly, the pathophysiological processes expressed in the brain such as neurotransmission anomalies, neuroinflammation and neurodegeneration, also occur in the retina in cases of neurodegenerative and psychiatric disorders (5). In view of all these developmental, anatomical and functional similarities between the retina and the brain, some authors claim that the retina is considered to be a window onto the brain and is relevant in neuroscience research (2).

Each layer of the retina has a specific importance in visual processing. The first layer is made up of photoreceptors, named rods and cones. Rods are specialized for night vision and spatial contrast whereas cones are responsible for daylight and color vision (6). The second layer is composed of bipolar cells, divided into cone bipolar cells and rod bipolar cells. Interneurons such as horizontal cells are also located between photoreceptors and bipolar cells (2). Other interneurons called amacrine cells may also act as local integrators and modulate the signal transmitted by bipolar cells to retinal ganglion cells (RGCs), which form the final layer of retinal neurons. RGCs are divided into M and P cells. M cells represent about 15% of retinal RGCs while P cells can represent up to 80% of all RGCs (7, 8). The distribution of M cells is mainly present in the peripheral retina, whereas the distribution of P cells is mainly present in the central retina and superimposed on that of the cones (9). Thus, P cells are sensitive to color while M cells are more specialized in contrast. These cells will give, respectively, the magnocellular and parvocellular pathways in the lateral geniculate nucleus LGN (10).

Interestingly, the phototransduction takes place in the retinal photoreceptor cells and this process enables us to study the electrical activity of the retina. For this, we can use electrophysiological methods such as an electroretinogram (ERG), which records the electrical response of different retinal layers. Such activity is recorded in response to the projection of visual stimuli such as flashes with the flash-ERG (fERG) or alternating black-and-white checkerboards with the pattern-ERG (PERG) (6). Use of this method sheds light on the physiological and pathological processes present in the retina. That is why the retina may offer an insight of what is happening downstream in the brain. One of its major advantages resides in the fact that the response of different retinal cells can be isolated from the fERG and the PERG. For instance, rod response can be explored with the Dark-Adapted 0.01 exam, using the b-wave (6). This procedure allows the projection of a sequence of 16 low intensity flashes in dark conditions in order to investigate in more detail the scotopic system, i.e., the rods and the associated bipolar cells (6). Similarly, the PERG examination is particularly suitable for investigating the N95 and the P50 waves. The N95 wave corresponds to a negative wave which appears at approximately 95 ms post-stimulation. The P50 wave corresponds to a positive wave which appears at approximately 50 ms post-stimulation. These waves correspond to the activity of the RGCs, notably with regards to the N95 wave (11).

The M and P RGCs project to the magnocellular and parvocellular layers which form the optic nerve. Both layers transmit visual information to the subsequent brain structure in the thalamus, the LGN, which is the first-order relay of visual information to the cortex (12). Unlike ERG, which targets the activity of various retinal layers, it is almost impossible to isolate the activity of the LGN in electrophysiology (13). However, LGN axons form neural projections called optic radiations, which create the connection between the LGN and the primary visual cortex (V1). Thus, V1 activity can be investigated using visual evoked potentials (VEP) in EEG (VEP-EEG) with the same stimuli as for ERG. Hence, electrophysiological biomarkers in the visual cortex focus on early components, such as P100 in VEP-EEG. This wave reflects primary visual characteristics such as brightness and contrast and also emphasizes V1 activity. By extension from the retina to the brain, this wave also makes a functional link with the RGCs and the N95 wave (14, 15).

**Figure 1:**
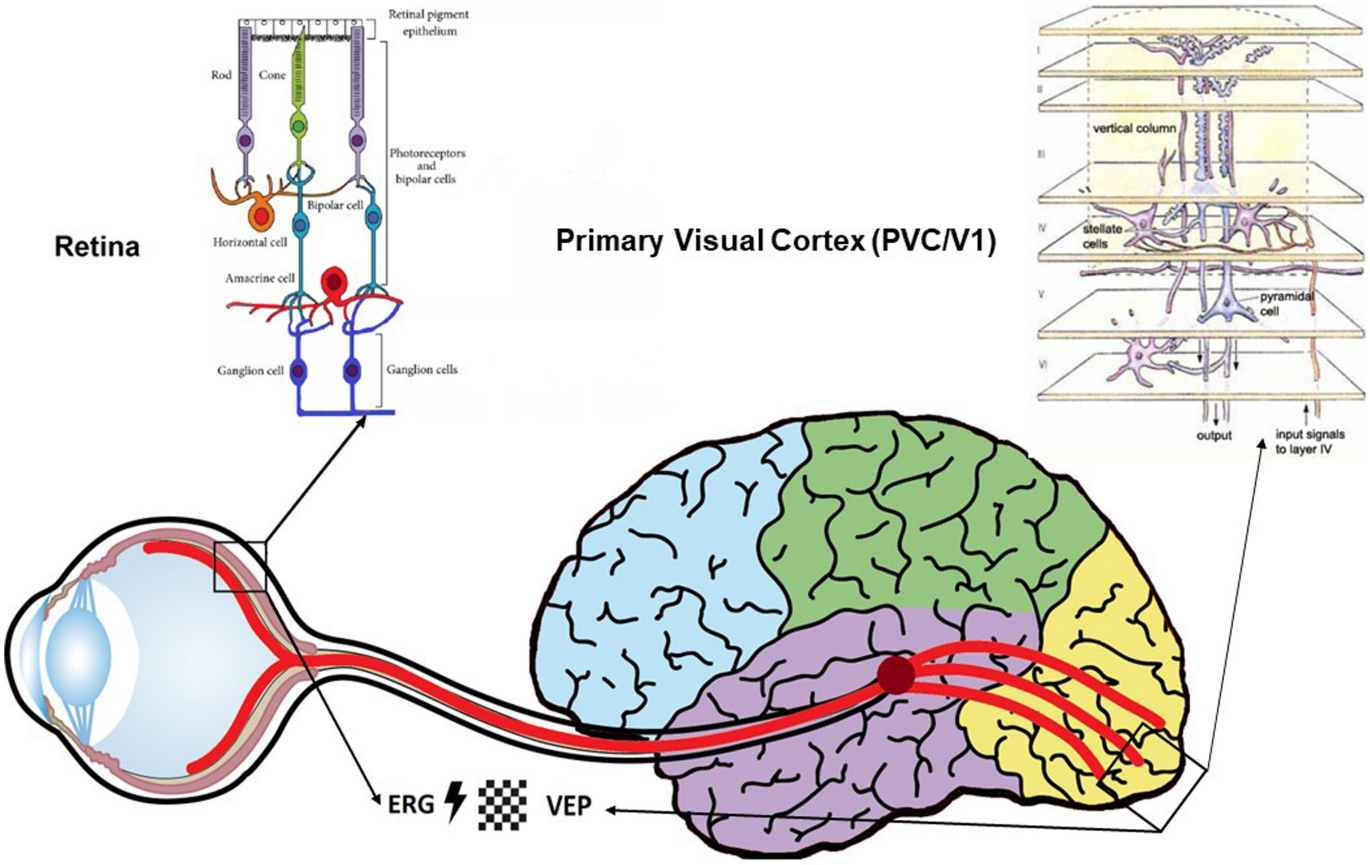
Schematic representation of the visual pathway highlighting the anatomical and physiological continuity between the retina and the brain, as well as the feasibility of functional exploration of these pathways using electrophysiological measurements (ERG/VEP-EEG). These two systems can be stimulated by the same types of visual stimuli as defined by the ISCEV international criteria (flashes and black-and-white checkerboard). Structural and functional similarities between the retina and the visual cortex encourage the investigation of electrophysiological visual indicators, which could have a use as relevant diagnostic biomarkers in psychiatric disorders.

Investigation of retinal and cortical electrophysiological biomarkers would appear to be a powerful tool for the diagnosis of neurodegenerative disorder such as Parkinson and Alzheimer diseases as well as psychiatric disorders such as schizophrenia and bipolar disorders. For instance, major retinal outcomes in psychiatric disorders showed that photoreceptor and retinal ganglion cell response in patients with neurodegenerative diseases such as Huntington’s disease, Alzheimer’s disease (AD) and schizophrenia was delayed compared to control subjects (16–18). Such results highlight a gradual reduction in the transmission of visual information in patients under both scotopic and photopic conditions (19). In the visual cortex, visual sensory processing during low-level visual tasks such as spatial frequency gratings, flashes and visual contour tasks has been highlighted in neurodegenerative disorder such as Alzheimer and psychiatric diseases such as schizophrenia and bipolar disorders with substantially reduced amplitude of the P100 wave (20–23). That is why, visual dysfunctions are considered as one of early symptoms in neurodegenerative diseases (24) and also in psychiatric disease (25). All in all, such results provide continuity between retinal and cortical abnormalities in neurodegenerative and psychiatric disorders and also bring to light relevant research perspectives regarding the potential value of combining several indicators in clinical research (Figure 1).

### 2.2. Potential value of gathering various visual indicators in clinical research

At present time, one of the major problems identified in routine clinical practice is the lack of objectivity in diagnosis. For this reason, the addition of new approaches such as electrophysiological indicators to routine clinical assessments facilitates diagnosis and ultimately improves data robustness *via* the development of additional and specific biomarkers of a psychiatric illness (1). Furthermore, electrophysiological visual indicators such as ERG and VEP-EEG offer a high level of objectivity, specificity and sensitivity (26). Moreover, they could provide a multitude of endophenotypes which, in addition to other techniques, would enable the creation of homogeneous subgroups of patients within a pathology or even the differentiation of one disease from another (27–29).

From a methodological and practical point of view, the use of electrophysiological indicators is relatively cost-effective, easy to record compared to other research methods, such as imaging, which often take up a lot of space and are of limited use in clinical practice. Moreover, they have the advantage of reflecting the objective measurement of normal biological or pathological processes, being valid, specific, and measured in a way that is easily reproducible between different categories of subjects (30). Although there is intra- and inter-individual variability, electrophysiological measurements benefit from a standardized methodology that allows the collection of similar plots between different subjects. Linked to the visual system, electrophysiology can map the flow of the visual signal extending from the retina to cortical regions. In short, both electrophysiological methods such as ERG and VEP-EEG are relevant because they reflect the neurophysiological underpinnings of pathophysiological processes, making it possible to isolate relevant indicators at the retinal and the visual cortical stages (31).

Such arguments underline that both ERG and VEP-EEG approaches could pave the way for creating new indicators in research, and hence contribute to the development of precision medicine in psychiatry. This would enable the creation of a personalized medical model and improve clinical decisions, treatments, or practices for subgroups of patients in place of a single medication model. In the future, one of the scientific and clinical challenges would be to contribute to the medical diagnosis of patients, which would allow, in addition to other preclinical signs, to make an earlier diagnosis (32).

For this purpose, our research focused on ERG and VEP-EEG measurements performed in neurodegenerative and psychiatric disorders. Indeed, there is a frequent transition from psychiatric disorders to neurodegenerative disorders. Moreover, the pathophysiological hypothesis of neurodegeneration remains within the realms of psychiatric conditions when many psychiatric symptoms are part of the clinical picture of neurodegenerative disorders. The literature mentions two methods for recording ERG and VEP-EEG. The first involves decoupling, which means recording first the ERG, then the VEP-EEG in the same subject with the same stimuli, for instance with flashes or black-and-white checkerboards. The second is a coupled approach, where both ERG and VEP-EEG are recorded simultaneously in the same participant with the same stimuli. The advantage of coupled measurements allows to investigate the continuity of the visual signal along the visual pathways from the retina to V1 and thus to precisely localize the deficits. Optionally, it is possible thanks to coupled measurements to isolate the time response between the retina and the visual cortex. That is why the goal of this present review is to discover the main indicators found in ERG and VEP-EEG, in both coupled and decoupled measurements and to determine the main components affected in neurodegenerative and psychiatric diseases. Finally, we will address the potential value of adding such measurements, in combination with advanced machine-learning algorithms, to routine clinical assessments with the aim of creating new biomarkers for clinical research.

## 3. Materials and methods

In order to thoroughly explore the literature on visual electrophysiological (retinal and cortical) indicators in psychiatric disorders, a search for relevant articles was conducted in the Pubmed and Google Scholar databases using the following keywords (“flash electroretinogram” OR “pattern electroretinogram” OR “retinal electrophysiological measurements” OR “electroencephalogram” OR “visual evoked potentials” OR “visual electrophysiology”) AND (“psychiatric disorders” OR “mental disorders” OR “schizophrenia” OR “psychotic disorders” OR “mood disorders” OR “bipolar disorders” OR “depressive disorders” OR “Alzheimer’s” OR “Parkinson’s disease”). All results up to April 1st, 2023, were examined for the selection process. Relevant publications were chosen *via* individual independent selection of titles by the authors: IR, KT, TS and VL. The articles selected had to be written in English and be related to the topic of the review. Additionally, a manual search was performed on the bibliography of each selected article.

## 4. Results

One of the first articles which investigated decoupled ERG and VEP-EEG techniques was Nightingale et al. (33) in 36 patients aged 63 years with Parkinson’s disease (PD) compared to 28 matched control subjects. Interestingly, the patients showed a significant reduction in PERG responses as reflected in the positive peak (PP1), which corresponds nowadays to the P50 ERG wave, and the second negative peak (PN2), which corresponds nowadays to the N95 ERG wave. Reductions were also found with regards to the P100 wave in VEP-EEG. Moreover, there was a significant inverse correlation between P100 latency and PERG amplitude independent of the age or sex of the patients. Thus, retinal disorders can affect the characteristics of the VEP-EEG at cortical level. According to the authors, PERG measurement may be particularly relevant to the investigation concerning a retinal origin of an abnormal VEP-EEG in PD (33).

Similarly, with coupled ERG and VEP-EEG measurements, Calzetti et al. (34) investigated the N95 ERG wave, the P50 ERG wave and the P100 VEP-EEG wave in 9 patients aged 64 years with early-stage PD of 6 months’ duration compared to 12 healthy controls. Stimuli were either transient PERG/VEP-EEG presented at different spatial and temporal frequencies. Authors showed a significant increase in PERG latency in the patients compared to the controls, with stimuli presented at 2.44c/d spatial frequency and 5.4 reversals per second. They also found reductions in VEP-EEG amplitudes and increased VEP-EEG latencies under the same condition between patients and controls. This VEP-EEG result was also found with transient and steady-state visual responses at different spatial frequencies. The authors suggested that PERG and VEP-EEG abnormalities can be highlighted in early-stage PD and appear to be stimulus-feature dependent. These changes in electrophysiological waves at both retinal and visual cortical level could provide a suitable basis for representing pathophysiological indicators of this neurodegenerative disorder (34).

With regards to Alzheimer’s disease, research using ERG and VEP-EEG is more recent. For instance, Krasodomska et al. (35) measured left eye activity using PERG and VEP-EEG measurements in 30 early-stage AD patients with a mean age of 73 years compared to 30 controls. Interestingly, they explored the retino-cortical time (RCT), which corresponds to the time difference between the cortical response and the retinal response. Firstly, patients displayed retinal ganglion cell dysfunctions, reflected by an increase in P50 ERG latency and reductions in P50 and N95 ERG amplitudes. Secondly, they reported an increase in P100 VEP-EEG latency as well as a significant increase in RCT in patients compared to controls (35). According to the authors, beta-amyloid and amyloid deposits in the retina and the optic nerve partly explain the cause of such electrophysiological deficits observed in Alzheimer’s patients. These deficits could also be provoked by deficits in retinal neurotransmitters, notably acetylcholine.

A more recent study by Ngoo et al. (36) investigated decoupled retinal and visual cortical electrophysiological measurements, studying right eye activity using PERG and VEP-EEG in 25 AD patients and 25 healthy controls. They also used optical coherence tomography (OCT) to investigate whether structural anomalies were present in the Retinal Nerve Fiber Layer (RNFL). Interestingly, the P50 ERG latency, the P50 ERG amplitude and the N95 ERG amplitude were correlated with RNFL thickness reduction in the average, superior and inferior quadrant in patients compared to controls. Results also indicated significant reduction of VEP-EEG amplitude and increased P100 and N130 VEP-EEG latencies in the visual cortex. According to the authors, the use of ERG and VEP-EEG combined with OCT would provide a better understanding of visual dysfunctions early in the visual pathway in AD patients (36).

In psychiatric disorders, only one study investigated both retinal and cortical electrophysiological measurements. This study was conducted by Bubl et al. (37) who investigated ERG and VEP-EEG responses in 40 patients with major depressive disorder (MDD) and 28 healthy controls. The analysis was performed not in the spatial domain but in the frequency domain, using a Fast Fourier Transform (FFT). The authors used checkerboard visual stimuli of 0.51° check size with 12.5 reversals per second (12.5 Hz) and a contrast of 3 to 80%. The results showed a reduction in VEP-EEG amplitude compared to healthy subjects. PERG amplitude and VEP-EEG amplitude were reduced to approximately 50 and 75%, respectively, in patients compared to controls. Interestingly, these results were correlated with the intensity of the depression, suggesting that deficits in electrophysiological measurements are correlated with the clinical symptomatology of the pathology, at both retinal and cortical level (37). Despite the relevant findings of this study, the frequency domain analysis poses a limitation for comparison with other results in the spatial domain.

Overall, the studies show increases in latency and decreases in amplitude, both in the ERG and in the VEPs. Indeed, we find decreases in P50 and N95 amplitudes at the retinal level and decreases in P100 amplitude at the cortical level in Parkinson’s and Alzheimer’s disease. All these results have been summarized in Table 1.

**Table 1:** Summary of the coupled and decoupled PERG and VEP-EEG results found in the literature for pattern stimulation.

Coupled (C) Decoupled (D)	Population	Results for patients compared to HC					Comments	Authors
		PERG		VEP – EEG				
		P50	N95	P100	N1	N135		
D	36 PD patients 28 HC	↓AMP	↓AMP	↓AMP ↑LAT	NA	NA	Inverse correlation between P100 latency and P50 amplitude/N95 amplitude	Nightingale et al. (33)
C	9 PD patients 12 HC	↑LAT	↑LAT	↓AMP ↑LAT	↓AMP ↑LAT	NA	↓VEP-EEG with transient and steady-state responses at different spatial frequencies	Calzetti et al. (34)
D	30 AD patients 30 HC	↓AMP ↑LAT	↓AMP	↑LAT	NA	NA	↑RCT in patients compared to controls	Krasodomska et al. (35)
D	15 AD patients 15 HC	↓AMP ↑LAT	↓AMP	↓AMP ↑LAT	NA	↓AMP ↑LAT	Combined OCT and ERG/VEP-EEG recording ↓RNFL thickness correlated with PERG measurements	Ngoo et al. (36)
D	40 MDD patients 28 HC	↓AMP (~75%)		↓AMP (~50%)			Fast Fourier Transform analysis (FFT) Checkerboards visual stimuli at High Frequency	Bubl et al. (37)

AD, Alzheimer’s Disease; PD, Parkinson’s Disease; MDD, Major Depressive Disorder; HC, Healthy Controls; FFT, Fast Fourier Transform; PERG, Pattern Electroretinogram; VEP-EEG, Visual Evoked Potentials in EEG; RCT, Retino-Cortical-Time; N, sample size; NA, non-Applicable.

## 5. Discussion

Using coupled and decoupled ERG and VEP-EEG studies, this review investigated which electrophysiological visual indicators are relevant in neurodegenerative and psychiatric diseases. Major results indicated deficits in both retinal and cortical measurements obtained using a decoupled technique. Such abnormalities are reflected in a decrease in PERG amplitude in the retina, such as in the N95 and the P50 ERG waves, and increased VEP-EEG latency in the visual cortex, such as in the P100 VEP-EEG wave, which could reflect retinal and cortical visual abnormalities. However, there are some factors that may influence the reproducibility of the electrophysiological waves.

### 5.1. Influence of several factors on electrophysiological parameters

#### 5.1.1. Neurodegeneration

Some pathophysiological mechanisms linked to the disease can be mapped in electrophysiological plots, thereby improving predictions about the disease. For example, a drop in the level of neurotransmitters initiates a neurodegenerative process and could have an incidence on the electrophysiological waves in psychiatric diseases (37–40). By way of illustration, electrophysiological alterations in AD are probably due to fibrillar neurodegeneration and the deposit of amyloid proteins, which causes degeneration of the optic nerve and therefore impacts the function of the RGCs. Parisi et al. (18) also argued that neural toxicity and RGCs dysfunction in ERG studies were mediated by abnormalities in neurotransmitters such as acetylcholine in AD. Similarly, PD is characterized by electrophysiological anomalies which can be caused by the destruction of dopamine neurons in the retina and the brain. The reverse is true where dopaminergic treatments such as L-Dopa can also influence retinal function (41–43). Such assumptions were also supported in dementia with Lewy body disease, where reduced a-wave and b-wave amplitude appears to be linked to a loss in dopamine cells (44). In sum, electrophysiological measurements could act as functional biosignatures for investigation of neurodegeneration and neural network activity (1).

#### 5.1.2. Medication

The influence of medication such as dopaminergic antagonists may have an impact on electrophysiological measurements and could be of use as a treatment response indicator in psychiatric diseases. Indeed, in schizophrenia, antipsychotics play an important role in the ERG waves, with studies such as that by Bernardin et al. (26) having pointed out the link between ERG parameters and dopaminergic implication. Similarly, dopamine treatment, in particular D2-class dopamine antagonists, could change the amplitude and suprathreshold of the components of the fERG exam such as the b-wave (45). In mice models, the knockout of dopaminergic type 1 and type 2 receptors indicated a decrease in b-wave amplitude (46). Interestingly, the model was developed to suggest the role of GABA (including GABA-dopamine interactions) in schizophrenia (47). Many studies also confirmed the role of genetic deletion of various receptors such as GABAR and their potential impact on the ERG components such as the b-wave (48). Similarly, glutamate is one of the main neurotransmitters involved in vertical retinal neurotransmission (49). In the psychiatric field, antipsychotics for schizophrenia emphasize glutamatergic treatment (50, 51), which may also have an impact on the ERG waves (52, 53).

However, these theories remain controversial since various studies have shown that alterations in the cone response and in other ERG waves were independent of medication or the dose of antipsychotic medication (42, 54, 55), particularly in the acute stage of the disease (56). Furthermore, other studies using dopaminergic antagonists, serotonergic antagonists and dopaminergic administration had no effect on ERG waves (57, 58), which could suggest that electrophysiological anomalies are present above and beyond the effect of medication (59).

#### 5.1.3. Variation factors on electrophysiological measurements

Although electrophysiological abnormalities were identifiable in psychiatric diseases, it is important to bear in mind that such findings could be influenced in a minor way by other factors unrelated to the pathology, which is also represent a limitation. Age or sex gender may be a consideration in the influence of these electrophysiological parameters (33). For instance, age might impact the perception of spatial frequency as early as the retina and upstream to the visual cortex (60) due to the loss of visual cells (61). Similarly, sex gender and notably hormones could influence retinal and cortical visual function (62–64).

### 5.2. Why is it important to add new indicators in clinical research?

Adding electrophysiological indicators such as ERG and VEP-EEG to routine medical assessments would improve the prognosis in psychiatric disorders. Furthermore, such measurements could enhance data robustness, improve the performance of current pathophysiological biomarkers, and increase our understanding of the localization and timing of potential electrophysiological deficits along the entire visual pathway. In short, finding strong biomarkers that will allow clinicians and psychiatrists to develop precision psychiatry will undoubtedly advance research (1, 2, 65).

Secondly, ERG and VEP-EEG visual dysfunctions would also exist in a more comprehensive spectrum of psychosis (66, 67) encompassing addictive substance use and subgroups of subjects at high risk for mental disorders (29, 68–70). Accordingly, adding further indicators such as coupled ERG and VEP-EEG measurements would predict vulnerabilities for later transition to a psychiatric disorder, particularly for bipolar disorders and schizophrenia (1, 71). Thus, the clinical applications of combined ERG and VEP-EEG methods are intended to highlight biomarkers of mental disorders, which will be useful in solving current clinical problems in terms of differential diagnosis, early detection, prediction of response to treatment, or clustering of clinical populations into homogeneous groups (72). One of the perspectives will be to orient the diagnosis towards electrophysiological signatures in mental health, while considering the patient’s care pathway in terms of personalized, predictive, participative, and preventive medicine. In fact, studies emphasize the strong link between the retina and the brain. Moreover, ophthalmologic manifestations often precede symptoms in the brain, indicating that eye investigations could offer a means of early diagnosis in many diseases (5). Coupled ERG and VEP-EEG methods could offer the possibility of strengthening the link between visual retinal and visual cortical measures, instead of recording these parameters alone. In addition, coupled ERG and VEP-EEG measurements could prevent the loss of important information between the retinal and cortical stages along the visual pathways. This point is in accordance with the study of Celesia et al. (61) and Krasodomska et al. (35) with regards to the RCT which could bring more precise information about the neuronal communication at the level of visual pathways between the retina and the visual cortex. Today, considerable and growing research efforts are therefore being made to improve current indicators and also to identify new ones in psychiatric illnesses.

In the future, the simultaneous acquisition of ERG and VEP-EEG measurements will undoubtedly provide additional information. This kind of combined data integrates both the very early retinal level as well as the primary visual brain areas in the occipital cortex. These two visual levels carrying different but complementary information on the same phenomena. Rather than such integrative treatment consisting of analyzing each modality, separately and combining the results at a second stage, it is more efficient to consider the data set as a whole to capture in full more complex relationships within the data. This could be resumed by the assumption of Aristotle who said that “the whole is greater than the sum of the parts.” Such fusion methodologies have already been proven to increase the sensitivity of the methods in various fields (73) and in particular in medical applications (74). In this perspective, the fusion of data acquired in response to different stimuli, each revealing different signatures of alterations in the system under examination, are likely to be helpful in discriminating disorders (75). Such advances are also possible thanks to signal processing and machine learning, which are tools of particular interest for the extraction of robust biomarkers from multimodal data, due to their ability to collate and combine information.

This review concerning coupled electrophysiological measurements has many strengths. To the best of our knowledge, this is the first review which summarized coupled and decoupled results of visual electrophysiological parameters in neurodegenerative and psychiatric diseases. Previously, only studies presenting ERG or VEP-EEG results alone were published. Second, although these methods are relatively old, the development of precision psychiatry on these measures may bring to light interesting new research perspectives regarding this current research topic in psychiatry. This review has also limitations. For instance, there were very few results regarding coupled or decoupled results and methodology associated to the results are outdated. In the same way, we noticed that some parameters can influence the electrophysiological measurements, but it also can be a strength by providing evidence of the effect of the medication treatments in various psychiatric disorders.

## 6. Conclusion

Electrophysiological measures related to the visual system are of particular interest for mental health, especially if added to other indicators in routine clinical assessments. The literature mainly showed deficits in N95 ERG wave and P100 VEP-EEG wave obtained with coupled and decoupled ERG/VEP-EEG measurements in PD, AD, and major depressive disorder. Although several factors — such as age, sex gender and medication — influence electrophysiological data, the present results reveal the challenge of including additional electrophysiological indicators associated with the visual system in clinical research. For instance, coupled ERG and VEP-EEG measurements with the help of machine learning techniques are also required to improve precision medicine and to accurately establish the location of abnormalities along the visual pathway. The advantages they offer will improve patient management in healthcare services and optimize the clinical diagnosis of various neurodegenerative and psychiatric pathologies such as PD, AD, schizophrenia and bipolar disorders to create precision medicine.

## Author contributions

TS, VL, VL-D, and RS were responsible for the research concept and design. KT, IR, HM-M, and GG participated in all bibliographic research. KT, IR, TS, and SC drafted the manuscript. All authors contributed to the article and approved the submitted version.

## Conflict of interest

KT, IR, and HM-M were employed by BioSerenity.

The remaining authors declare that the research was conducted in the absence of any commercial or financial relationships that could be construed as a potential conflict of interest.

## Publisher’s note

All claims expressed in this article are solely those of the authors and do not necessarily represent those of their affiliated organizations, or those of the publisher, the editors and the reviewers. Any product that may be evaluated in this article, or claim that may be made by its manufacturer, is not guaranteed or endorsed by the publisher.
